# Clinical and metabolomic characterization of Brivanib‐Induced hypertension in metastatic colorectal cancer

**DOI:** 10.1002/cam4.6248

**Published:** 2023-06-17

**Authors:** Jodi I. Rattner, Karen A. Kopciuk, Hans J. Vogel, Patricia A. Tang, Jeremy D. Shapiro, Dongsheng Tu, Derek J. Jonker, Lillian L. Siu, Chris J. O'Callaghan, Oliver F. Bathe

**Affiliations:** ^1^ Cumming School of Medicine Arnie Charbonneau Cancer Institute, University of Calgary Calgary Alberta Canada; ^2^ Department of Mathematics and Statistics, Faculty of Science University of Calgary Calgary Alberta Canada; ^3^ Department Biological Sciences, Faculty of Science University of Calgary Calgary Alberta Canada; ^4^ Department of Surgery and Oncology, Cumming School of Medicine University of Calgary Calgary Alberta Canada; ^5^ Department of Medical Oncology Carbini Hospital Melbourne Victoria Australia; ^6^ Department of Community Health & Epidemiology Queens University Kingston Ontario Canada; ^7^ Division of Medical Oncology Ottawa Hospital Cancer Centre Ottawa Ontario Canada; ^8^ Division of Medical Oncology and Hematology Princess Margaret Cancer Centre Toronto Ontario Canada

**Keywords:** cancer biomarker, chemotherapy, colorectal cancer, hypertension, metabolomics, survival

## Abstract

**Background:**

Trials of tyrosine kinase inhibitors (TKI) have not demonstrated dramatic benefits in advanced colorectal cancer (CRC), and this may be a function of poor patient selection. TKI‐induced hypertension is reportedly a surrogate marker for treatment benefit for some tumor types. Our objective was to determine whether hypertension was associated with benefit in the context of CRC treatment, and also to gain insight on the pathogenesis of TKI‐induced hypertension by monitoring associated changes in the circulating metabolome.

**Patients and Methods:**

Clinical data were acquired from clinical trial patients with metastatic CRC randomized to cetuximab ± the TKI brivanib (*N* = 750). Outcomes were evaluated as a function of treatment‐induced hypertension. For metabolomic studies, plasma samples were taken at baseline, as well as at 1, 4, and 12 weeks after treatment initiation. Samples were submitted to gas chromatography–mass spectrometry to identify treatment‐related metabolomic changes associated with TKI‐induced hypertension, compared to pre‐treatment baseline. A model based on changes in metabolite concentrations was generated using orthogonal partial least squares discriminant analysis (OPLS‐DA).

**Results:**

In the brivanib treated group, 95 patients had treatment‐related hypertension within 12 weeks of initiating treatment. TKI‐induced hypertension was not associated with a significantly higher response rate, nor was it associated with improved progression‐free or overall survival. In metabolomic studies, 386 metabolites were identified. There were 29 metabolites that changed with treatment and distinguished patients with and without TKI‐induced hypertension. The OPLS‐DA model for brivanib‐induced hypertension was significant and robust (R^2^Y score = 0.89, Q^2^Y score = 0.70, CV‐ANOVA = 2.01 e‐7). Notable metabolomic features previously reported in pre‐eclampsia and associated with vasoconstriction were found.

**Conclusion:**

TKI‐induced hypertension was not associated with clinical benefit in metastatic CRC. We have identified changes in the metabolome that are associated with the development of worsening brivanib‐induced hypertension that may be useful in future efforts of characterizing this toxicity.

## 1 INTRODUCTION

Colorectal cancer (CRC) is the second most common cause of cancer‐related mortality worldwide. Over the last two decades, there have been advancements leading to an expansion of the therapeutic armentarium, leading to improved patient outcomes. Multi‐kinase anti‐angiogenic receptor tyrosine kinase inhibitors (TKIs), which represent important agents for a number of malignancies, are now also established therapeutic options for metastatic CRC. For example, regorafenib is associated with improved survival.^1^, ^2^ However, response rates are not dramatic, limiting their impact. A number of other TKIs have been submitted to large clinical trials, with mixed degrees of benefit.^3^ The limited benefit of TKIs in combatting metastatic CRC may be a product of patient selection. Unfortunately, there are no predictive biomarkers currently available that aid in patient selection, and therefore trials will naturally include individuals who will not have the attributes required to benefit. As a result, the apparent benefit found in large clinical trials would be diminished.

A number of published reports have suggested that TKI‐induced hypertension represents a clinical biomarker or surrogate for benefit. Better survivals have been reported in patients with TKI‐induced hypertension compared to patients who did not develop hypertension in renal cell carcinoma, well differentiated thyroid cancer, gastrointestinal stromal tumors, and hepatocellular carcinoma.^4^, ^5^, ^6^, ^7^ This has not been studied in the context of CRC. CO.20 was a negative randomized phase III trial comparing cetuximab plus placebo versus cetuximab and brivanib, a TKI targeting vascular endothelial growth factor and fibroblast growth factor, in metastatic, chemotherapy‐refractory, KRAS wild‐type CRC.^8^ We sought to determine whether TKI‐induced hypertension confers a benefit.

Simultaneously, we wanted to explore any potential changes in the circulating metabolome in order to derive an improved understanding of the pathogenesis of TKI‐induced hypertension. The metabolome has been intensively investigated in hypertensive conditions such as preeclampsia.^9^ Clinically, TKI‐induced hypertension shares some features with preeclampsia, including the frequent co‐existence of proteinuria and edema, and so it is possible that there are some commonalities in their pathogenesis.^10^, ^11^

## 2 METHODS

### 2.1 Patients

This study was approved by the Health Research Ethics Board of Alberta Cancer Committee (HREBA‐CC 14–0074). Data and samples used were derived from an international, multicenter, double‐blind, randomized controlled phase III study examining the benefits of adding brivanib to third‐line cetuximab (NCIC‐CTG CO.20) in patients with KRAS wild‐type metastatic CRC who progressed on combinations of a oxaliplatin, irinotecan and fluoropyrimidine.^8^ Patients were randomized to cetuximab and brivanib (*N* = 376) or cetuximab and placebo (*N* = 374). In that trial, the addition of brivanib to cetuximab did not have a demonstrable benefit.^8^ Patients with uncontrolled hypertension at baseline (systolic blood pressure > 150 and diastolic blood pressure > 100 mmHg) were ineligible for the trial.

**FIGURE 1:**
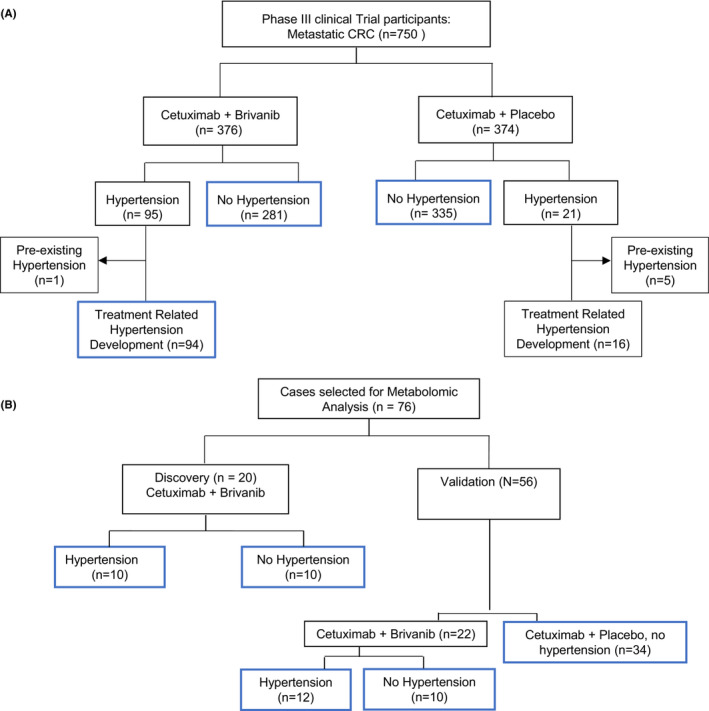
A Consort diagram depicting patient and data flow used in the analyses. Blue boxes indicate comparator groups/samples. (A) Consort diagram representing comparison of clinical characteristics and outcomes associated with brivanib‐induced hypertension. (B) Sample selection for metabolomic biomarker discovery and validation.

Brivanib‐associated hypertension was defined as new onset hypertension after starting the drug, or an exacerbation of hypertension following treatment initiation. In the experimental arm (cetuximab plus brivanib), cases with treatment‐related hypertension (*N* = 95) were compared to patients without hypertension (*N* = 281). Patients from the cetuximab plus placebo treatment arm who did not have treatment‐related hypertension acted as controls (Figure 1A). Patients with hypertension as a comorbidity were excluded from calculation of survival outcomes.

### 2.2 Clinical monitoring and blood samples

In accordance with Common Terminology Criteria for Adverse Events (CTCAE) (version 3.0), the appearance and severity of hypertension were graded at baseline, Week 1, then every 4 weeks until tumor progression. Briefly, the CTCAE grades for hypertension are defined as follows: Grade 1 hypertension is defined as a transient increase in diastolic blood pressure > 20 mmHg (diastolic) or a total blood pressure > 150/100; Grade 2 hypertension is defined as recurrent or symptomatic increases in diastolic blood pressure > 20 mmHg with the potential requirement of additional monotherapy; and finally, Grade 3 hypertension is defined as a recurrent increase in diastolic blood pressure > 20 mmHg in combination with a required administration of one or more therapeutic interventions.

Blood was collected at baseline, 2 weeks, 4 weeks and then every 4 weeks until disease progression. Plasma for metabolomic analysis was collected at baseline (prior to treatment initiation), and during the first 12 weeks after treatment (Weeks 1, 4, and 12) in K2‐EDTA lavender top tubes (Thermo Fisher Scientific). Patients were not required to fast. Samples were processed within 2 h of collection, then stored at −80°C prior to analysis. For metabolomic studies, 76 patients were studied (Figure 1B). This included 10 patients with brivanib‐related hypertension and 10 patients without hypertension in a discovery cohort; as well as a validation cohort consisting of 22 patients from the brivanib arm (12 with hypertension, 10 without), and 34 controls from the cetuximab alone arm.

### 2.3 Gas chromatography–mass spectrometry

Gas chromatography–mass spectrometry (GC–MS) was performed as previously described.^12^ Briefly, metabolite extraction was based on the methods of Bligh and Dyer.^13^ Derivatization was performed using methoxyamine‐hydrochloride in pyridine solution, and the silylating agent N‐Methyl‐N‐(trimethylsilyl) trifluoroacetamide (MSTFA; Millipore‐Sigma). Samples were diluted using hexane. Spike‐in internal standards consisted of a mix of deuterium‐labeled metabolites representative of diverse chemical classes with a range of retention indices (phenylalanine D‐5, d‐glucose‐D7, malonic acid‐D4, glycine‐D5, palmitic acid‐D31, l‐leucine‐D10, l‐lysine‐D9, and myo‐inositol‐D6 at concentrations at the mid‐range of the linear part of each of their standard curves).

A Bruker Scion 436 GC–MS (Bruker Daltonics Inc) was used at a MS range of 50–800 m/z. Serial plasma samples from each patient were deliberately included in the same batch, but randomly distributed. Batches were designed to include approximately equal representation of age group, treatment arm, and sex. Calibration standards consisted of aliphatic alkanes (n‐decane, n‐docosane, n‐dodecane, n‐hexacosane, n‐nonadecane, n‐pentadecane, n‐triacontane; Millipore‐Sigma). Additionally, pooled quality controls were distributed throughout each batch, prior to each series of 10 experimental samples. Reproducibility of the quality control samples was analyzed based on variability of abundance of internal standards. Variability within and between batches was within an acceptable range, <20%.

### 2.4 Data analysis

Data normality and homoscedasticity were confirmed using the Shapiro–Wilks test and the Breush–Pagan test, respectively. Group comparisons over time were performed using a two‐way repeated measures ANOVA using IBM SPSS Statistics (version 27, International Business Machines Corporation). Progression‐free survival (PFS) was calculated from the time of treatment initiation until radiographic criteria of disease progression were observed. Overall survival (OS) was calculated from the time of initiation of treatment until patient death. Survival curves were estimated using the Kaplan–Meier method, then compared using the log‐rank test using GraphPad Prism (version 7.0, GraphPad Software Inc).

Mass spectra were processed, quantified, and analyzed using Metabolite Detector software (Version 2.06, Technische Universität Carolo‐Wilhelmina zu Braunschweig). Sample peaks were normalized utilizing the spiked‐in internal controls. That is, intensities of the internal standard metabolites were integrally normalized against the sum of the metabolite intensities for each sample, adjusting for inter‐sample variations in concentrations. Metabolites were identified referencing two in‐house feature libraries (GOLM metabolite data base and NIST), with reference to retention indices, retention times and individual ions. A second normalization step was performed using median fold‐change methods. Missing values were imputed with the minimum quantitative value in the data set. Batch‐dependent noise was removed, and inter‐batch variation was corrected using the ComBat algorithm (through the Bioconductor R package “sva”) in R environment (version 3.3).

Changes in metabolite abundance levels from baseline were calculated for each post‐treatment time point. Treatment‐related perturbations at each time point were compared. Data analysis was performed using SIMCA‐P+ software (version 15.0, Umetrics AB). Data structure was explored by principal component analysis (PCA) and outliers were removed. Subsequently, a supervised analysis was performed using orthogonal partial least squares discriminant analysis (O‐PLS‐DA). Candidate metabolite selection was based on variable importance on projection (VIP) thresholds set to maximize R^2^Y and Q^2^Y values and to minimize the difference between them, as previously described.^13^ These scores were also used for further assessment of multivariate model performance, and dataset variance covered by the model was evaluated in a 7‐fold cross‐validation.

### 2.5 Pathway analysis

Knowledge‐based pathway analysis methods were employed to derive a more complete understanding of the functional context of metabolites that were perturbed with hypertension. Metabolites that were differentially altered from baseline as a function of hypertension were initially submitted to MetaboAnalyst (Version 4.0, https://www.metaboanalyst.ca) in order to identify perturbed metabolic pathways from its HMDB‐derived archives. Chemical KEGG identifiers were generated from network analyses, which were subsequently manually examined to derive potential effects on metabolic pathways, using a knowledge‐based approach.

## 3 RESULTS

### 3.1 Incidence of hypertension and clinical correlates

Table 1 summarizes the prevalence and severity of hypertension over the first 12 weeks of treatment in each treatment arm. By Week 12 hypertension was observed in 95 patients (34.4%) in the cetuximab + brivanib arm and 21 patients (5.3%) in the control arm (cetuximab + placebo). Brivanib‐associated hypertension was apparent by the first week of treatment in all cases. Time of onset of hypertension was seen to be much more variable tn the cetuximab‐only treatment arm. Table 2 describes the clinical characteristics of patients who did and did not develop hypertension. We could not identify any distinguishing features associated with treatment‐related hypertension. Proteinuria was more common in hypertensive patients in both arms of the trial, although that was only statistically significant in the cetuximab only arm.

**TABLE 1:** Incidence of each grade of hypertension in each treatment arm over the first 12 weeks of treatment.

Hypertension grade	Cetuximab + Brivanib			Cetuximab + Placebo		
	Treatment Week 1	Treatment Week 4	Treatment Week 12	Treatment Week 1	Treatment Week 4	Treatment Week 12
0	317 (84.3%)	328 (87.2%)	281 (74.7%)	359 (95.9%)	359 (95.9%)	353 (94.4%)
1	6 (2.0%)	13 (3.5%)	13 (3.5%)	1 (0.3%)	2 (0.5%)	5 (1.3%)
2	39 (10.4%)	20 (5.3%)	56 (14.9%)	11 (2.9%)	10 (2.8%)	14 (3.7%)
3	14 (3.7%)	15 (4.0%)	25 (6.6%)	3 (0.8%)	3 (0.8%)	2 (0.5%)
4	0	0	1 (0.3%)	0	0	0

**TABLE 2:** Clinical characteristics of patients with and without treatment‐related hypertension, in each treatment arm.

		Cetuximab + Brivanib (*N* = 376)		Cetuximab + Placebo (*N* = 374)	
		Hypertension (*n* = 106)	No hypertension (*n* = 269)	Hypertension (*n* = 21)	No hypertension (*n* = 353)
Sex					
	Male	65 (61.3%)	182 (67.7%)	16 (76.2%)	218 (61.8%)
	Female	41 (38.7%)	87 (32.3%)	5 (23.8%)	135 (38.2%)
Age		62.6 ± 1.3	62.4 ± 1.3	65.8 ± 4.0	62.4 ± 1.1
ECOG					
	0	43 (40.5%)	70 (26.0%)	8 (38.1%)	117 (33.1%)
	1	53 (50.0%)	170 (63.2%)	12 (57.1%)	201 (56.9%)
	2	10 (9.5%)	29 (10.8%)	1 (4.8%)	35 (10.0%)
Site of primary Tumor					
	Colon	54 (50.9%)	154 (57.2%)	12 (57.1%)	218 (61.8%)
	Rectum	34 (32.1%)	86 (32.0%)	8 (38.1%)	37 (10.4%)
	Colon and Rectum	18 (17.0%)	29 (10.8%)	1 (4.8%)	98 (27.8%)
Site of Disease					
	Liver	86 (81.1%)	202 (75.1%)	16 (76.2%)	268 (75.9%)
	Lung	66 (62.2%)	180 (66.9%)	14 (66.7%)	211 (59.8%)
	Lymph	43 (40.5%)	114 (42.4%)	9 (42.9%)	143 (40.5%)
	Ascites	7 (6.6%)	51 (19.0%)	1 (4.8%)	47 (13.3%)
# of sites of disease					
	1	22 (20.8%)	44 (16.4%)	4 (19.0%)	67 (19.0%)
	2–3	65 (61.3%)	159 (59.1%)	20 (95.2%)	203 (57.5%)
	4^+^	19 (17.9%)	66 (24.5%)	4 (19.0%)	83 (23.5%)
# of Regimens					
	0	102 (96.2%)	258 (95.9%)	21 (100.0%)	338 (95.8%)
	1	4 (3.8%)	11 (4.1%)	0 (0.0%)	15
Proteinuria					
	0	99 (93.4%)	265 (98.5%)	20 (95.2%)***	350 (99.2%)***
	1^+^	7 (6.6%)	4 (1.5%)	1 (4.8%)***	3 (0.8%)***

*Note*: For each data point, data are expressed as *N* (%).

*** Statistically significant difference in proteinuria levels between patients in cetuximab+placebo arm who developed hypertension compared to those that did not (*p* > 0.0001).

Pre‐existing hypertension was apparent in one patient (0.3%) in the cetuximab+brivanib arm; their grade remained stable throughout treatment. There were five patients (1.3%) in the cetuximab only arm who demonstrated pre‐existing hypertension; in two patients with Grade 1 hypertension, their condition became worse (becoming Grade 2) during treatment. Incidence and severity of hypertension during treatment is summarized for each treatment arm in Table 1.

**FIGURE 2:**
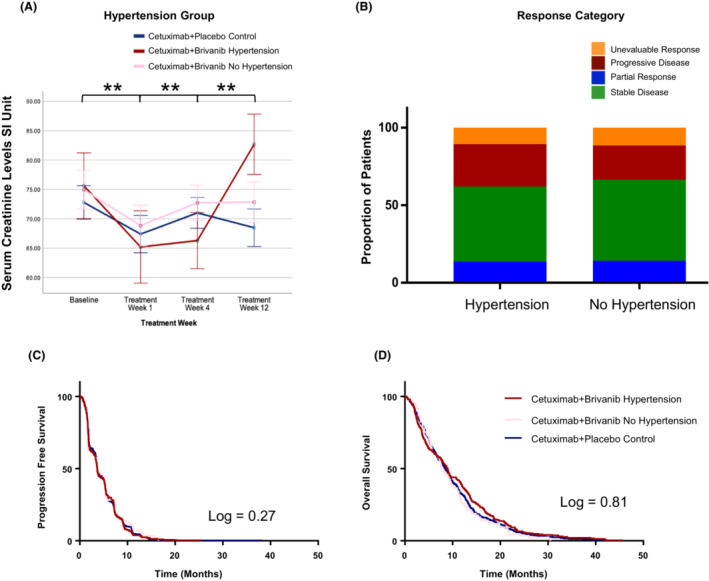
(A) Time‐related changes in serum creatinine levels in patients who received cetusimab + placebo, and in the brivanib + cetuximab arm who did and did not have treatment‐related hypertension. (B) Stacked bar graph comparing response categories in brivanib group. (C) Kaplan–Meier curve comparing progression‐free survival (PFS) in patients treated with brivanib + cetuximab (as a function of treatment‐related hypertension) and cetuximab + placebo controls. (D) OS as a function of treatment‐related hypertension in the brivanib + cetuximab arm, as well as controls.

Sodium, potassium, calcium, and magnesium levels did not differ as a function of hypertension or treatment group. Creatinine levels in all patients was seen to decrease shortly after treatment was initiated. In patients with brivanib‐associated Grade 2 or 3 hypertension, creatinine levels significantly increased by 12 weeks (Figure 2A). This pattern did not occur in the absence of hypertension or in the cetuximab‐only treatment arm.

In the brivanib‐treated arm, response rate based on RECIST 1.0^14^ criteria was evaluated for a potential association with hypertension status. A partial response (PR) was recorded in 13 patients (13.7%) with TKI‐induced hypertension and 40 patients (14.2%) who did not have hypertension (not significantly different). There was also no significant difference in the proportion of patients who had stable disease (Figure 2B). Treatment‐related hypertension was also not associated with improved PFS or OS, regardless of grade (Figure 2C,D). When compared with survival in the cetuximab + placebo treatment arm, there was no significant difference between groups (OS: 8.9 months for cetuximab + brivanib with hypertension, 7.8 months for cetuxmab + brivanib with no hypertension, 8.4 months in the cetuximab + placebo arm; PFS: 3.65, 3.71, and 3.68 months).

### 3.2 Brivanib‐induced changes in the circulating metabolome associated with hypertension

**FIGURE 3:**
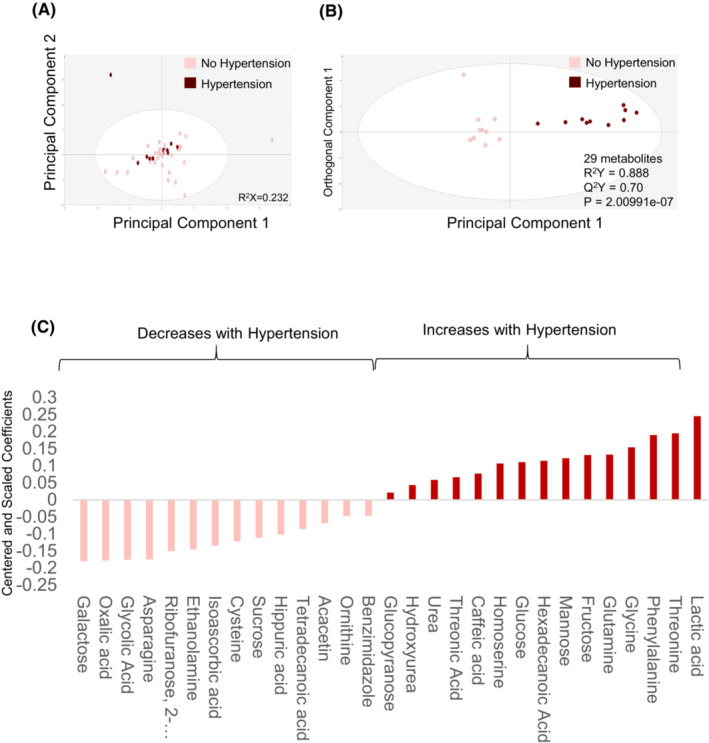
Changes in circulating metabolites are identifiable at 12 weeks after chemotherapy initiation. (A) PCA scatter plot depicting changes in plasma metabolites as a function of hypertension development. B. Supervised (OPLS‐DA) scores scatter plot based on a model distinguishing changes in the circulating metabolome that accompany treatment‐induced development of hypertension. C. Coefficient column plot describing directionality of changes of individual metabolites in association with chemotherapy‐induced hypertension. Metabolite correlations with the model are determined by Centered and Scaled (CS) coefficients. D. Loadings column plot depicting behavior of individual metabolites in the cetuximab control arm and the brivanib‐induced hypertension.

**FIGURE 4:**
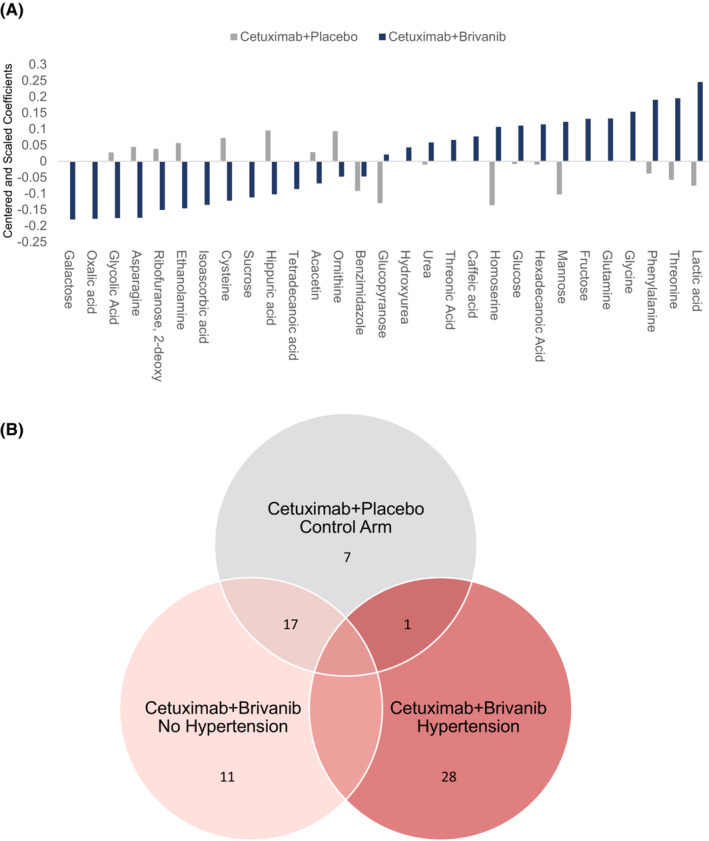
Changes in circulating metabolites associated with brivanib induced hypertension. A. Coefficient column plot describing the behavior of the biomarker in the two treatment arms. The pattern of metabolite changes observed in the brivanib arm were absent or opposite in directionality in the control arm. B. Venn diagram depicting the number of metabolites shared between each sub group of patients.

To determine how brivanib‐induced hypertension affects the circulating metabolome, changes in plasma metabolomic features were compared from baseline to 12 weeks after treatment initiation in patients with Grade 2 or worse hypertension and in patients without hypertension. GC–MS detected 386 common features in all samples, of which 94 were named metabolites and included in the downstream analysis. A preliminary PCA of 14 patients with hypertension and 56 patients without hypertension did not demonstrate any intrinsic pattern distinguishing the two groups, but two outliers were identified (outside of the 95% ellipse), which were excluded from further analysis (Figure 3A). Treatment‐related changes in metabolites were compared in 10 patients with hypertension and 10 patients without hypertension who were age and sex matched using O‐PLS‐DA. After filtering using a threshold of a VIP >1 and *p* < 0.05, a stable parsimonious model consisting of 29 metabolites was produced that distinguished the two groups (R^2^Y score = 0.89, Q^2^Y score = 0.70, CV‐ANOVA = 2.01 e‐7) (Figure 3B). The changes in metabolite concentrations relative to baseline at Week 12 are summarized in the coefficient column plot (Figure 3C) and Table 3. Figure 4A demonstrates that the treatment related metabolite changes differed in the two treatment arms. Moreover, Figure 4B demonstrates that the metabolomic perturbations related to brivanib‐related hypertension are unique, with the exception of benzimidazole.

**TABLE 3:** Metabolite changes from baseline to Week 12 post‐drug initiation, in patients who received brivanib and cetuximab. Metabolites listed comprised the multiparametric model that described the pattern of changes associated with treatment‐related hypertension. *p*‐Values were calculated using a Welch's *t*‐test.

Group comparison	Increased in treatment‐induced hypertension				Decreased in treatment‐induced hypertension			
	Metabolite	*p*	Fold change (HT)	Fold change (No HT)	Metabolite	*p*	Fold change (HT)	Fold change (no HT)

	Lactic acid	0.02	1.176	−0.098	Galactose	0.04	−1.517	0.960
	Threonine	0.01	3.130	−0.408	Oxalic acid	0.02	−0.761	0.041
	Phenylalanine	0.13	1.855	−0.675	Glycolyic acid	0.18	−0.725	1.056
	Glycine	0.11	0.379	−0.147	Asparagine	0.06	−0.282	0.012
	Glutamine	0.01	1.295	−0.068	Ribofuranose, 2‐deoxy	0.19	−0.192	0.247
	Fructose	0.28	0.065	−0.008	Enthanolamine	0.09	−0.539	1.077
	Mannose	0.02	4.754	−0.504	Isoascorbic acid	0.07	−0.304	0.375
	Hexadecanoic acid	0.12	0.625	−1.442	Cysteine	0.11	−0.625	0.119
	Glucose	0.14	4.144	−0.699	Sucrose	0.03	−0.261	0.205
	Homoserine	0.09	0.169	−0.213	Hippuric acid	0.01	−0.896	0.482
	Caffeic acid	0.15	0.486	−0.806	Tetradecanoic acid	0.48	−0.309	0.068
	Threonic acid	0.33	2.167	−0.469	Acacetin	0.07	−0.731	0.561
	Urea	0.05	0.249	−0.302	Ornithine	0.03	−0.591	0.147
	Hydroxyurea	0.14	2.378	−0.819	Benzimidazole	0.02	−0.640	0.030
Glucopyranose	0.01	0.939	−0.024				

Abbreviation: HT, hypertension.

**FIGURE 5:**
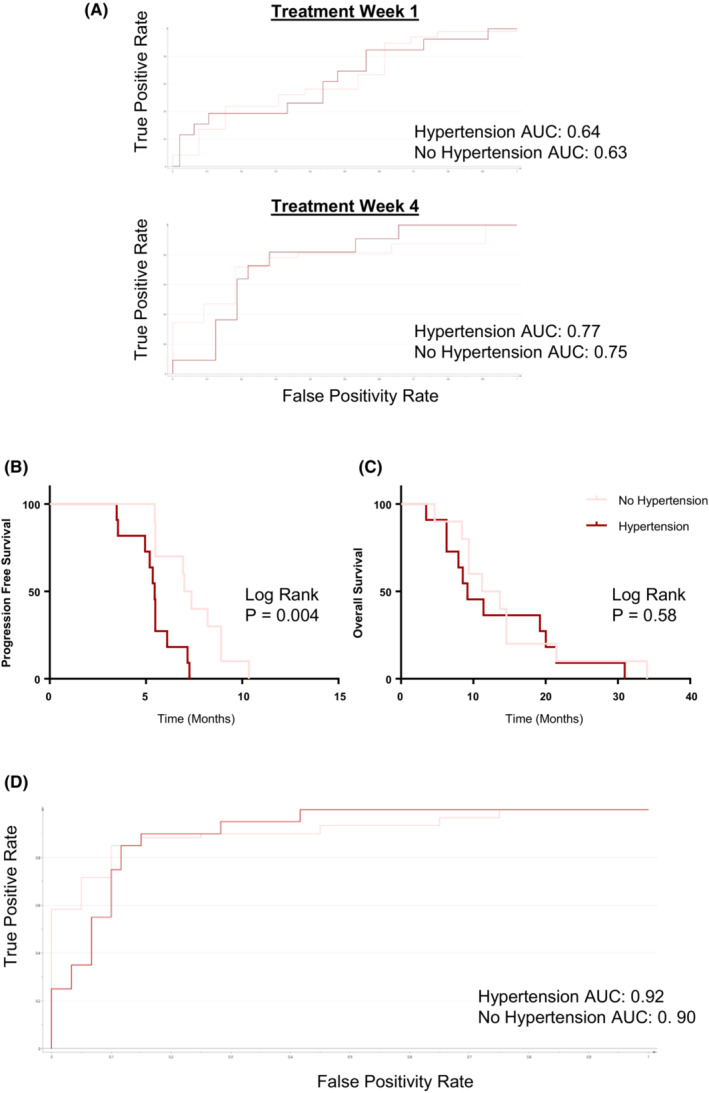
Biomarker‐defined hypertension vs. no hypertension, based on S‐scores. A. ROC curves for validation of metabolomic biomarker of hypertension at Weeks 1 and 4, in the independent validation cohort. B. Kaplan–Meier curves comparing progression‐free survival (PFS) in discovery patients with metabolomic biomarkers of treatment‐induced hypertension and no hypertension. C. Kaplan–Meier curves comparing overall survival (OS) in patients with metabolomic biomarkers of treatment‐induced hypertension and no hypertension. D. Validation ROC curve measuring performance of the metabolomic biomarker for hypertension/no hypertension in the validation cohort, Week 12.

A similar approach was taken at earlier time periods (Weeks 1 and 4) to determine whether systemic metabolomic changes appear at earlier time points (Figure 5A). Based on *R* ^2^ and *Q* ^2^ values, we were unable to develop satisfactory and stable models related to the presence of hypertension at these earlier time periods. The behavior of individual metabolites that comprised the hypertension‐related model from the 12 week time point were also examined. It was expected that we would see metabolic changes in all 29 metabolites constituting the hypertension biomarker earlier than 12 weeks. However, only a few of the metabolites manifested these changes at earlier time points. Mannose increased by Week 1 (*p* = 0.021). By Week 4, lactic acid and glutamine were increased (*p* = 0.025 and 0.011, respectively); benzimidazole, hippuric acid, and oxalic acid were decreased (*p* = 0.015, 0.01, and 0.027, respectively). No other significant changes in the remaining metabolites of the marker for hypertension were seen until Week 12.

To further assess the identified hypertension biomarker, we evaluated its association with PFS and OS (Figure 5B,C). In patients who had biomarker‐defined hypertension, PFS was significantly and markedly worse than those who did not have the biomarker. Specifically, PFS was 5.4 months when the hypertension biomarker was present, and 7.2 months when the hypertension biomarker was absent (*p* = 0.004). OS was not found to be significantly different between the two patient types based on presence of the hypertension biomarker.

Internal validation of the metabolomic signature of hypertension for CRC was performed in an independent cohort consisting of patients from the same clinical trial (*N* = 56) (Figure 5D). The hypertension biomarker was compared to CTCAE assigned grades of hypertension at Week 12. Overall, the hypertension signature had a favorable sensitivity of 92%, and a specificity of 90%. The biomarker for hypertension did not appear prior to the 12‐week time point in the validation cohort, similar to the discovery cohort.

### 3.3 Metabolic pathways perturbed in brivanib‐associated hypertension

Perturbed metabolites associated with TKI‐induced hypertension were submitted to pathway analysis to devise an understanding of the metabolic processes that drove our biomarker. Hypertension was associated with an increase in glucose, glucopyranose (the pyranose form of glucose), fructose, and mannose. While the elevated levels of glycolysis constituents could be a function of impaired glycolysis, they may also be a function of increased substrate availability. Lactic acid was notably increased in the absence of accelerated glycolysis.

TKI‐induced hypertension had a distinct pattern of amino acid perturbations. The amino acids phenylalanine, serine, and threonine were elevated in hypertensive patients, while levels of asparagine and cysteine were decreased. TKI‐induced hypertension was associated with increased levels of urea, hydroxyurea, and glutamine. Increased conversion of glutamine to urea is thought to inhibit synthesis of arginine. Ornithine and asparaginine, precursors of arginine, were at low levels.

Finally, increased levels of free fatty acid (FFA) hexadecanoic acid were found in association with TKI‐induced hypertension.

## 4 DISCUSSION

TKIs are becoming more commonly used for a number of malignancies. In the context of metastatic CRC, bevacizumab and regorafenib have established efficacy,^1^, ^15^ but response rates are not dramatic. Trials of sorafenib, sunitinib, and brivanib treatments did not demonstrate benefit in patients with metastatic CRC.^4^, ^6^, ^7^, ^8^, ^16^ The marginal benefit of TKIs in CRC may be partly due to the inability to select individuals who will benefit. In the absence of a predictive biomarker, it has been suggested that the appearance of hypertension is a surrogate for TKI effectiveness.^17^ If this were the case, then a short course could be administered to identify the subgroup most likely to benefit. Hypertension is a significant (but not usually dose‐limiting) toxicity associated with multi‐kinase anti‐angiogenic TKIs. In one retrospective study, one‐third of patients developed new‐onset hypertension in follow up after chemotherapy.^18^ The incidence increases to over 50% in patients on TKIs.^11^, ^19^

The appearance of hypertension after initiating TKIs is reportedly associated with greater benefit in hepatocellular carcinoma, thyroid cancer, GIST, and renal cell carcinoma.^5^, ^19^, ^20^, ^21^, ^22^ We were interested in whether the development of TKI‐induced hypertension would cause a similar beneficial effect in CRC patients. Contrary to the other tumor types, we found that brivanib‐associated hypertension did not confer higher response rates, or an improved PFS or OS. This may be due to several factors. The appearance of hypertension may have resulted in dose reductions. This was a third‐line chemotherapy trial, and the benefits of brivanib may not be as apparent in heavily pretreated patients. Finally, it is possible that brivanib did not target a clinically meaningful biological pathway.

Brivanib is a dual inhibitor of VEGF and FGF signaling pathways.^23^ The appearance of hypertension suggests that there is effective VEGF inhibition, as its effects on blood pressure are primarily mediated by VEGF inhibition, which activates nitric oxide synthase.^24^, ^25^ For that reason, it would be expected that hypertension would predict greater therapeutic efficacy. On the other hand, in CRC, VEGF inhibition by itself has only modest activity. In the CO.20 trial, despite effective VEGF inhibition, there was no measurable survival benefit over EGFR inhibition alone. The benefit of bevacizumab is only realized when administered in combination with cytotoxic chemotherapy,^26^ and even then it is not a dramatic benefit. Aflibercept, a recombinant fusion protein targeting VEGF‐A, VEGF‐B, and placental growth factor, did not improve survival in combination with FOLFOX in the first line setting,^27^ and only improved OS from 12.1 to 13.5 months in a second line study in which it was given in combination with FOLFIRI.^28^ Ramuciramab, which targets VEGFR2, similarly had only modest benefit when given in combination with FOLFIRI.^29^ As we have found, in a phase II trial examining the relationship between bevacizumab, hypertension, and clinical outcomes in CRC, Feliu et al. found that there was no significant association between hypertension and response rate, PFS or OS.^30^ Therefore, VEGF inhibition, which is primarily responsible for TKI‐induced hypertension, may have limited impact on CRC biology.

Clinical bloodwork in our study demonstrated an association of brivanib‐associated hypertension with increased serum creatinine levels. While a decline in renal function has been reported in patients on TKIs,^31^ long term renal function is generally unaffected.^32^ Elevated creatinine levels are known to be a frequent accompaniment of TKI administration.^31^ It is generally thought that TKI‐associated increases in serum creatinine reflect renal dysfunction secondary to the vasoconstrictive properties of VEGF inhibition.^33^ Structural changes in glomeruli and thrombotic microangiopathy have also been reported.^34^ However, creatinine is not only secreted in urine by glomerular filtration; it is also actively secreted across epithelial cells via transporters, which can account for a significant proportion of total creatinine clearance. Recent reports have shown that TKIs inhibit creatinine transport, suggesting that elevated creatinine levels are not necessarily indicative of renal failure.^35^

In order to gain an understanding of the pathogenesis of TKI‐induced hypertension, we also performed an exploratory metabolomic analysis. While large numbers of samples were not available for validation, some consistent metabolomic perturbations were observed. The experimental design involving serial samples represented a powerful approach to identifying treatment‐related changes in the metabolome. There were alterations in the circulating amino acid profile that are known to affect vascular physiology. For example, serine and threonine reportedly exert their influence on blood pressure via activation of the sympathetic nervous system and increasing the thickness of blood vessels by increasing their vascular tone.^36^, ^37^ Phenylalanine is known to bind and activate the calcium‐sensing receptor of vascular smooth muscle cells, inducing vasoconstriction.^38^ Changes seen in urea, ornithine, and asparagine are associated with reduced arginine bioavailability. l‐arginine is a potent vasodilator, and arginine deficiency is known to increase vasoconstriction and endothelial cell dysfunction.^39^ High levels of hexadecenoic acid were seen in TKI‐induced hypertension. Increased polyunsaturated fatty acids, including hexadecanoic acid, seen in hypertensive rats, are thought to increase neurovascular tone by inhibiting endothelial nitric oxide synthase.^40^, ^41^, ^42^ Finally, high lactate levels seen with hypertension may be due to peripheral vasoconstriction.^43^

One interesting feature of brivanib‐associated hypertension was the increased levels of monosaccharides, including glucose, fructose, and mannose. This may partially be a result of impaired glycolysis. Indeed, in a study on the hepatotoxicity associated with TKIs, Paech et al. showed that TKIs are mitochondrial toxicants that inhibit glycolysis.^44^ What is unclear is why galactose levels are decreased in brivanib‐associated hypertension. Galactose is converted to glucose‐6‐phosphate mostly in the liver, entering the glycolytic pathway by the Leloir pathway. If glycolysis were indeed impaired, one might expect galactose levels to rise.

Numerous studies have been performed on the metabolomic features accompanying preeclampsia.^9^ TKI‐induced hypertension shares some clinical features with preeclampsia, including frequent concomitant edema, hypertension, proteinuria, and hypoalbuminemia.^10^ Interestingly, some of the metabolomic features accompanying brivanib‐associated hypertension have been reported in preeclampsia. Odibo et al. observed increased levels of alanine, phenylalanine, and glutamate.^45^ Mukherjee et al. reported elevated glutamate, alanine, and decreased arginine.^46^ Kenny et al. also found higher levels of in glutamate.^47^ Decreased asparagine levels have been observed in preeclampsia.^48^ These similarities are intriguing, as they may point to some commonalities in their pathogenesis.

In conclusion, we found that the development of hypertension in CRC patients undergoing TKI administration was not associated with a survival benefit as seen in some other tumor types. Using GC–MS, we were able to identify a pattern of treatment‐related changes in the plasma metabolome that accompany hypertension. While the metabolomic studies were exploratory in nature, they have some common pathogenic features seen with preeclampsia. Further research is required to validate and confirm the identified metabolic profile.

## AUTHOR CONTRIBUTIONS

**Jodi I. Rattner:** Data curation (lead); formal analysis (lead); investigation (lead); methodology (lead); validation (lead); visualization (lead); writing – original draft (lead); writing – review and editing (lead). **Karen A. Kopciuk:** Methodology (supporting); supervision (equal); writing – review and editing (equal). **Hans J. Vogel:** Methodology (equal); resources (lead); supervision (equal); writing – review and editing (equal). **Patricia A. Tang:** Supervision (equal); writing – review and editing (equal). **Jeremy D. Shapiro:** Data curation (equal); project administration (equal); writing – review and editing (equal). **Dongsheng Tu:** Data curation (equal); project administration (equal); resources (equal). **Derek J. Jonker:** Conceptualization (equal); data curation (equal); project administration (equal). **Lillian L. Siu:** Conceptualization (lead); data curation (lead); funding acquisition (equal); investigation (equal); project administration (lead); writing – review and editing (equal). **C J O'Callaghan:** Conceptualization (equal); data curation (lead); funding acquisition (lead); investigation (equal); project administration (lead); writing – review and editing (equal). **Oliver F. Bathe:** Conceptualization (lead); formal analysis (lead); funding acquisition (lead); investigation (lead); methodology (equal); project administration (equal); resources (equal); software (equal); supervision (lead); validation (lead); writing – original draft (equal); writing – review and editing (lead).

## CONFLICT OF INTEREST STATEMENT

Authors declare no potential conflict of interest.

## ETHICS STATEMENT

This study was approved by the Ethics Committee of the Foothills Medical Centre of the University of Calgary (HREBA.CC‐14‐0074). All participants provided written informed consent.

## Data Availability

The authors of this paper have provided the original data to support the conclusions of this paper without unnecessary reservations.
